# Morphological and Chemical Changes in the Trophozoites and Cysts of *Acanthamoeba Castellanii* Induced by *Camellia Sinensis* Extracts

**DOI:** 10.1007/s11686-024-00941-9

**Published:** 2025-03-03

**Authors:** Lenu B. Fakae, Jizhou Zhong, Ka Lung Andrew Chan, Subbareddy Mekapothula, Gareth W. V. Cave, Xing-Quan Zhu, Carl W. Stevenson, Hany M. Elsheikha

**Affiliations:** 1 School of Veterinary Medicine and Science, University of Nottingham, Loughborough, LE12 5RD UK; 2 School of Biosciences, University of Nottingham, Loughborough, LE12 5RD UK; 3 Institute of Pharmaceutical Science, King’s College London, London, SE1 9NH UK; 4 School of Science and Technology, Nottingham Trent University, Nottingham, NG11 8NS UK; 5 College of Veterinary Medicine, Shanxi Agricultural University, Taigu, 030801 Shanxi China; 6 Department of Animal Science, Faculty of Agriculture, Rivers State University, Nkpolu-Oroworukwo P.M.B. 5080, Port Harcourt, 500101 Rivers State Nigeria

**Keywords:** *Acanthamoeba castellanii*, *Camellia sinensis*, FTIR microspectroscopy, Nuclear integrity, Nucleic acid

## Abstract

**Purpose:**

*Acanthamoeba castellanii* is an important opportunistic human protozoal pathogen that can cause both skin, ocular and brain infections. Recent studies have established that brews and solvent extract (SE) of green tea (*Camellia sinensis*) can inhibit the growth and encystation of *A. castellanii*. Here we characterized those growth and encystation inhibitions.

**Methods:**

Herein, we characterize of the morphological and chemical changes that occur in the trophozoites and the encysting stage of *A. castellanii* after exposure to *C. sinensis* SE and brew using Transmission electron microscopy (TEM), Fourier-transform infrared (FTIR) microspectroscopy and fluorescence-based assays.

**Results:**

TEM showed ultrastructural changes in both *A. castellanii* stages. FTIR microspectroscopy revealed modifications of amide I and II band peaks in the *C. sinensis*-treated trophozoites, suggesting an inhibition of protein synthesis. Assessment of the nucleus integrity of trophozoites exposed to SE and brew revealed disruption of the nuclear membrane integrity, nuclear fragmentation, and chromatin degradation, and reduction in the quantity of DNA and RNA, indicating trophozoite death. These results are consistent with *C. sinensis* acting as a membrane-active anti-acanthamoebic, exhibiting amoebicidal activity against growing and encysting *A. castellanii*. This work underlines the importance of characterizing the effect of *C. sinensis* constituents, individually or in combinations, to clarify which ones are the primary components responsible for its action and the observed alterations in the structure and function of *A. castellanii*.

**Conclusion:**

These results demonstrated that exposure to *C. sinensis* SE or brew alters the synthesis of protein, DNA, RNA and disrupts the cell wall integrity.

**Supplementary Information:**

The online version contains supplementary material available at 10.1007/s11686-024-00941-9.

## Introduction

*Acanthamoeba castellanii* is an important parasitic agent that can cause a wide range of serious health conditions, including granulomatous amoebic encephalitis [1], *Acanthamoeba* keratitis (AK) [2–4], and inflammation of the lungs and skin [5]. Polyhexamethylene biguanide (PHMB) and chlorhexidine (Chx) digluconate are the treatments of choice for *Acanthamoeba* infections [6, 7]. Because of the inadequate therapeutic effects of monotherapy with PHMB or Chx, their usage has involved combination with diamidines; propamidine isethionate or hexamidine [6, 8, 9]. However, the risks of treatment failure and relapse are high [10], primarily due to the lack of efficacy against *Acanthamoeba* cysts.

The limitations of current therapies for AK warrant the development of improved treatment approaches. To identify novel alternative treatments, we recently examined the anti-acanthamoebic activity of green tea (*Camellia sinensis*; *C. sinensis*) and demonstrated that *C. sinensis* brews and solvent extract (SE) possess strong inhibitory activity against the growth and encystation of *A. castellanii* trophozoites [11, 12]. We have also demonstrated the inhibitory effect of ( −)-epigallocatechin-3-gallate (EGCG), a major com-ponent of *C. sinensis*, on *A. castellanii* growth [13]. In another study, we investigated the activity of the solvent extract (SE) of *C. sinensis* and its main chemical ingredients against the trophozoites and cysts of *A. castellanii*. Results showed that *C. sinensis* SE inhibited trophozoite replication and encystation and caused cytolysis and the disruption of mem-brane integrity of trophozoites. Interestingly, *C. sinensis* SE was not toxic to human corneal epithelial cells up to 2500 µg/mL [12].

Our previous study identified six chemical constituents (caffeine, EGCG, epicatechin gallate, kaempferol, quercetin, and theogalline) in *C. sinensis* brews [11] and ten constituents (caffeine, catechin, epicatechin, epicatechin gallate, EGCG, epigallocatechin, kaempferol, myricetin, theobromine, and theogallin) in *C. sinensis* SE [12]. *C. sinensis* brew [11] and SE [12] had shown significant effects on *A. castellanii*, however, their mechanism of actions remains unknown. Given that more chemical constituents were identified in *C. sinensis* SE compared with the brew form, it is important to investigate whether the brew and SE have different effects on the phenotypic features and chemical structure of *A. castellanii*. In this study, we characterized the ultrastructural cellular and nuclear changes that occur in trophozoites and cysts of *A. castellanii* after exposure to *C. sinensis* SE and brew. We also determined the changes in the chemical constituents in trophozoites in response to *C. sinensis* SE and brew using Fourier-transform infrared spectroscopy (FTIR) microspectroscopy.

## Materials and Methods

### Parasite Strain

**Fig. 1:**
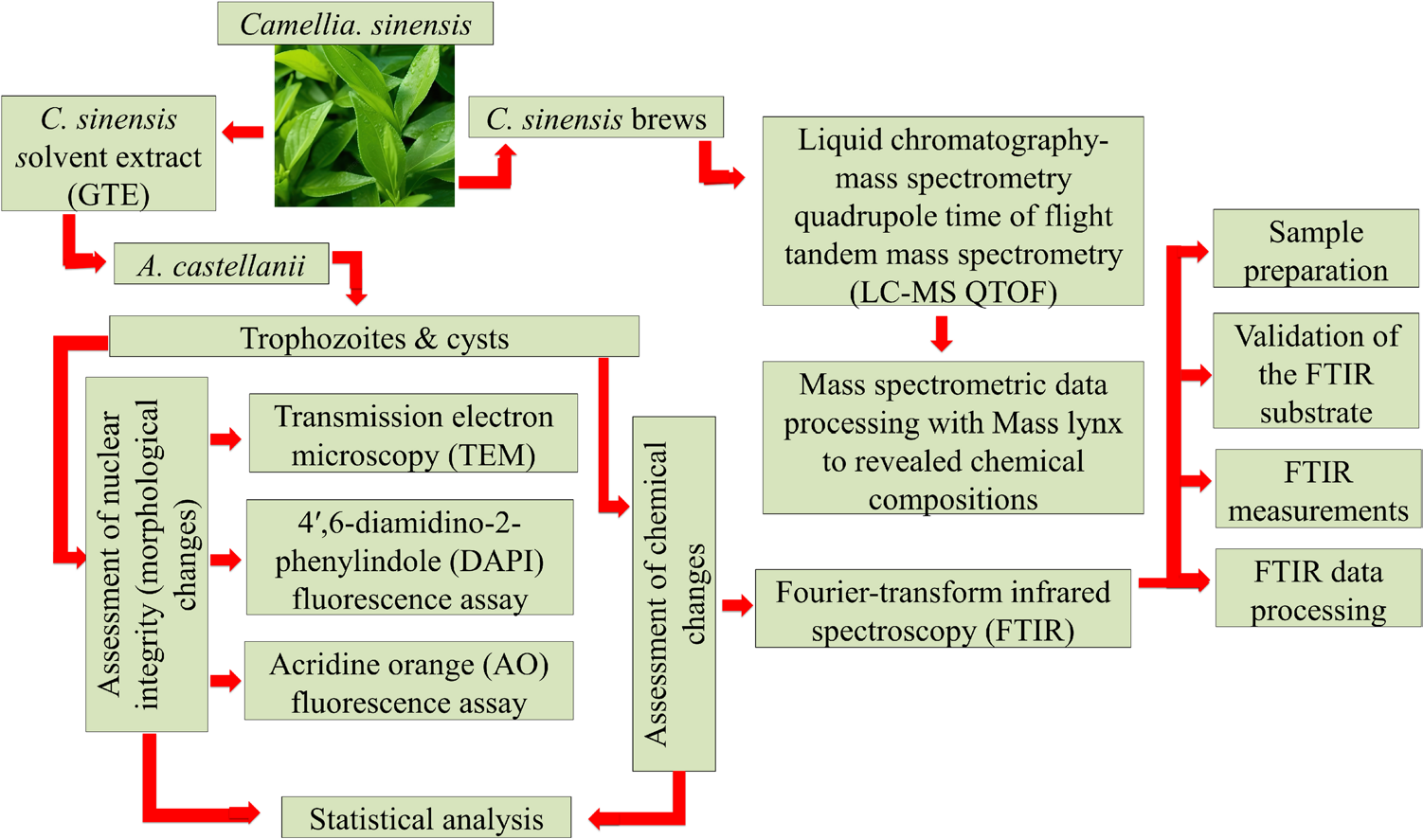
Schematic representation of the experiments conducted in this study

The *A. castellanii* strain of the T4 genotype (American Type Culture Collection; ATCC 30011) was used and maintained in peptone -glucose-yeast (PGY) medium at 25 °C as described previously (Fig. 1) [11].

### Preparation of C. sinensis Forms

The solvent extraction of *C. sinensis* and preparation of solvent extract (SE) solutions for testing were per-formed as described previously [12]. The preparation of *C. sinensis* brew was described previously [11].

### Liquid Chromatography-Mass Spectrometry Quadrupole Time of Flight Tandem Mass Spectrometry (LC–MS QTOF) Analysis of C. sinensis Forms

The *C. sinensis* forms, including cold brew, hot brew, and acetonitrile extract were dissolved in solvent A (5 mL) and sonicated for 10 min at room temperature. The extracts were filtered via syringe filters (0.24 µm). Subsequently, the extract solution (1 mL) was placed in amber LC–MS vials for the LC–MS analysis. LC–MS analysis was performed using Waters Acquity UPLC hyphenated with Xevo G2-XS QTOF (Waters, Manchester, UK). Chromatographic conditions were as follows: Solvent A—water + 0.1% formic acid; Solvent B—acetonitrile + 0.1% formic acid at a flow rate of 9.25 mL min − 1, runtime 13 min on RP-C18 UPLC column (Restek Raptor^®^ 2.7 µm, 100 × 2.1 mm at 30 °C). Chromatographic Gradient elution: Solvent B—3% ramped to 95% in 9.5 min; and isocratic to 11 min and re-equilibrated to 3% at 13 min. The mass spectrometry was calibrated for high resolution and set to scan from 100 to 1500 Daltons with a scan speed of 0.1 s. The electrospray ionization was performed in a positive mode with a capillary voltage of 2.5 kV, sampling cone of 24 kV, and source offset of 80 kV. The source temperature and desolvation were set to 120 and 300 °C while cone and desolvation gas flow were set up to 20 and 800 L/hr. Mass spectrometric data were processed using Mass Lynx to identify the individual active chemical components from *C. sinensis* forms.

### Transmission Electron Microscopy (TEM)

The *A. castellanii* trophozoites and cysts treated with *C. sinensis* SE or brew in respective experiments were fixed in 3% glutaraldehyde in 0.1 M cacodylate buffer for 24 h. Further processing of the samples was performed strictly as described previously [11]. TEM micrographs were obtained with a JEM-2100Plus TEM (JOEL, Tokyo, Japan) and micrographs were taken using a Rio16 camera with Digital Micrograph 3 (Gatan, Pleasanton, California, United States).

### Effects of C. sinensis SE and Brew on Nuclear Integrity of Trophozoites

We used two fluorescence-based assays, based on staining using 4′,6-diamidino-2-phenylindole (DAPI) and acridine orange (AO), to determine to which degree *C. sinensis* SE influenced the nuclear integrity of trophozoites within 24 h exposure compared with the control. In both assays, trophozoites were treated with 1250 µg/mL *C. sinensis* SE, 50% brew, PGY (negative control), and Chx (positive control). DAPI-treated trophozoites emit blue fluorescence when the nucleus of viable trophozoites absorb the dye. This fluorescence is visible at an excitation peak of 359 nm and an emission peak of 457 nm. At 24 h post-treatment, trophozoites were washed with PBS and fixed with 4% paraformaldehyde (PFA) for 10 min. The fixed trophozoites were washed twice with PBS and suspend-ed in permeabilizing buffer (4% PFA and 0.5% Triton X-100) for 10 min. The permeabilized trophozoites were washed with bi-distilled water 3 times to remove the permeabilizing buffer and resuspended in 200 μL of bi-distilled water and two drops of the DAPI stain were added, and the sample incubated for 15 min. A few drops of the stained trophozoites suspension were dropped on a microscope glass slide, covered with a coverslip and the edges were sealed with transparent nail polish. Slides were viewed under an inverted Zeiss LSM 710 confocal micro-scope (Zeiss, Germany) running on ZEN 2011 software (Carl Zeiss microscopy, Germany). Bright field, phase contrast and fluorescence images were obtained. The images were analyzed using Carl Zeiss microscopy software (Zen Blue, version 3.3, Germany), and the ImageJ (Fiji) software (Maryland, United States).

The AO stain was also used to detect nuclear changes (DNA and RNA) of trophozoites in response to treatment compared with the control groups. AO is a cell-permeant nucleic acid binding dye that emits green fluorescence (by intercalation) when it binds to double stranded DNA and orange fluorescence (by electrostatic attractions) when it binds to single-stranded DNA or RNA. Viable trophozoites stained with AO (Thermofisher Scientific, UK) fluoresce green and orange under fluorescent microscopy when the excitation wavelength range is switched between 500⁄526 for DNA and 460⁄650 for RNA. Following the removal of treatment medium, trophozoites were washed, fixed, and stained with 1% AO for 15 min protected from light. The stained trophozoites were washed 3 times in bi-distilled water, and the pellets were resuspended in 100 µL of PBS. A few drops of AO-stained trophozoite suspension were casted on a microscope slide, covered with a coverslip and the edges were sealed with transparent nail polish. Images were obtained using an inverted Zeiss LSM 710 confocal microscope (Zeiss, Germany) with Rhodamine (red for RNA) and FITC (green for DNA) fluorescence filters.

### FTIR Spectroscopic Analysis

FTIR microspectroscopy was used to analyze the chemical changes that occurred in trophozoites in response to treatment with *C. sinensis*. FTIR is a non-invasive analytical technique that utilizes infrared light to monitor changes in the chemical components of organisms by tracking changes in absorbance patterns produced following treatment [14–16].

#### Sample Preparation

To characterize the effect of *C. sinensis* on trophozoites, ~ 3.2 × 105 trophozoites were seeded in T25 cm2 tissue culture flasks. The flasks were treated with 1250 µg/mL SE or 50% hot brew. Negative and positive control flasks included trophozoites treated with PGY medium alone and PGY medium + 0.02% Chx, respectively. After 24 h and 48 h, the trophozoite suspension was harvested from each flask and transferred to falcon tubes, then washed twice with PBS and once in bi-distilled water by centrifugation at 1204 xg for 5 min. After centrifugation, the trophozoite pellets were fixed in 4% PFA for 24 h. Before measurement, trophozoite suspension was centrifuged as above to remove the PFA and washed twice in bi-distilled water and stored in isotonic saline solution at 40 °C until use. Before the spectral measurement, the samples were mounted on a 1 mm thick CaF2 window by drop casting followed by gentle rinsing in bi-distilled water briefly to remove any remaining salt crystals and then thoroughly air-dried for 30 min. The trophozoites and cysts were found to be dispersed individually across the substrate when prepared in this way.

#### Validation of the FTIR Substrate

FTIR measurement of single trophozoites often produces a strong scattering baseline effect when the shape of cells is round and, especially, when the size of the cells is a similar length scale as the wavelength of mid-IR light [17]. In this study, the size of the fixed was 10–15 µm and their largely round shape can limit obtaining high quality FTIR spectra from single trophozoites with CaF2 substrate. Therefore, we investigated the suitability of the substrate used in the FTIR microspectroscopy imaging before using it for the main experiments. To achieve this purpose, we performed FTIR measurements of 10 randomly selected *A. castellanii* trophozoites treated with 1250 µg/mL *C. sinensis* SE using a flat CaF_2_ substrate versus ZnS hemispheres. This chemical fingerprinting analysis revealed a marked improvement in the baseline distortion with ZnS hemispheres (Supplementary Fig. 1). Therefore, all subsequent FTIR measurements were made using the ZnS hemisphere lenses. Previous studies have also showed that a combination of ZnS hemispheres and FTIR imaging approach can suppress scattering effects and increase magnification and spatial resolution of measurement [18].

#### FTIR Measurements

Single cell spectra were collected using a FTIR microscope (SpotLightTM 400, Perkin Elmer, USA) combined with ZnS hemispheres as previously described [19, 20]. Trophozoites were prepared by drop-casting on the flat surface of an upside-down ZnS hemisphere (32 mm radius, Crystran Ltd, Dorset, UK), and were left to dry at room temperature. After adherence of the trophozoites to the ZnS surface they were briefly rinsed gently twice with deionized water to remove the salt crystals. The trophozoites on the ZnS hemisphere were allowed to dry in air for 30 min. Another identical ZnS hemisphere was then placed on top of the upside-down ZnS hemisphere with a 12 µm spacer. The two hemispheres were held together with a lens-holder (Thorlab Ltd, Ely, UK) before centering on the microscope stage for single-cell analysis. The microscope aperture size was set to 30 µm × 30 µm, which is equivalent to ~ 13 µm × 13 µm (with 2.25 × through the ZnS hemispheres). A clean area with no trophozoites was used as the background location. The spectral resolution was set at 4 cm-1 with a spectral range of 4000–800 cm^−1^ and the number of scans was 64, with ap-proximately 1 min to acquire each spectrum. Measurement of 35 to 37 randomly selected individual trophozoites were performed for the control and treated trophozoites.

#### FTIR Data Processing

Spectrum software (PerkinElmer, Shelton, USA) was used to perform interactive poly-normal baseline correction with baseline points at 4000, 3725, 2380, 1800, 940, and 825 cm^−1^, followed by vector normalization calculated using Microsoft Excel^™^ (Microsoft Corporation, USA). The PyChem software version 3.0.5 g Beta (Python Software Foundation, Wilmington, Delaware, USA) was then used to perform principal component analysis (PCA) on both the absorbance spectra and the second derivative spectra. Microsoft Excel^™^ was used to calculate the p-values to compare between the control and the *C. sinensis* SE treated trophozoite samples.

### Statistical Analysis

All statistical analyses were performed using GraphPad Prism Version 9.0.0 (GraphPad Software Inc., CA USA). One-way and two-way analysis of variance (ANOVA) with Tur-key’s multiple comparison test were used to compare groups. The results are expressed as mean ± standard error of mean (SEM). *P* < 0.05 was considered statistically significant. For MIC values, cytotoxicity tests and selectivity index to determine 50% inhibition of cell growth expressed as 50% cytotoxic concentration (CC_50_) have been established in previous studies [11, 12]

## Results

### Chemical Analysis of C. sinensis Forms Using LC/MS‑QTOF

LC–MS QTOF profiling of *C. sinensis* (cold brew, hot brew, and acetonitrile-methanol extract (SE)) revealed 6 chemical compositions for the hot and cold brew and confirmed the same 10 chemical constituents detected in the SE previously [12]. Given the similarity in the composition of cold and hot brew, we opted to characterize the effect of hot brew only compared with *C. sinensis* SE.

### Ultrastructural Features of Treated Trophozoites

**Fig. 2:**
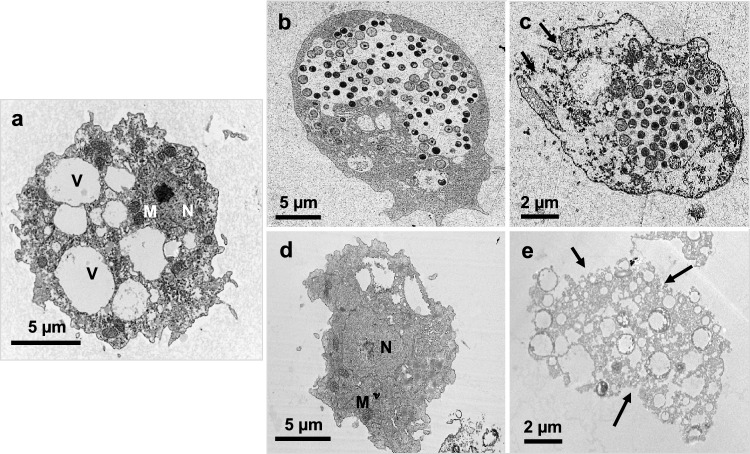
Representative TEM micrographs showing progressive destruction of *A. castellanii* trophozoites exposed to *C. sinensis* SE and brew. **a** Trophozoite in control PGY medium; **b** Trophozoite in 2500 µg/mL SE at 24 h, showing loss of acanthopodia and rounding up as they encyst to protect itself from the effect of *C. sinensis* solvent extract (SE). **c** At 72 h post-exposure to *C. sinensis* SE, trophozoite exhibited rupture of the plasma membrane (arrows). **d** Trophozoite treated with *C. sinensis* brew at 24 h shows loss of the cellular membrane integrity. **e** Exposure to *C. sinensis* brew for 72 h caused considerable loss of the cellular membrane integrity and depletion of the cytoplasmic contents. *V* food vacuole, *M* mitochondria, *N* nucleus

TEM micrographs confirmed the progressive destruction of trophozoites exposed to 2500 µg/mL of *C. sinensis* SE (Fig. 2b–c) and brew (Fig. 2d–e) compared to untreated trophozoites (Fig. 2a). At 24 h post-exposure, there was loss of acanthopodia and rounding of trophozoites, presumably to encyst to counter the stress caused by *C. sinensis* SE (Fig. 2b). Trophozoites treated with *C. sinensis* brew also showed loss of acanthopodia, with the reduction and/or loss of intracellular organelles such as mitochondria, food vacuole and the normally centrally located nucleus (Fig. 2d). At 72 h post-exposure to *C. sinensis* SE, there was distinctive loss of cellular membrane integrity and expulsion of the cytoplasmic contents, suggesting death of the trophozoites. We also observed condensation of the mitochondria, an undefined nucleus (i.e., rupture of nuclear membrane) in the treated trophozoites (Fig. 2c). Trophozoites treated with *C. sinensis* brew also showed distinctive loss of the cellular membrane integrity, blotting out of the food vacuoles and mitochondria, and depletion of the cytoplasmic contents (Fig. 2e). At 72 h, 1250 µg/mL of *C. sinensis* SE shown similar characteristics to 2500 µg/mL *C. sinensis* SE. Non-treated trophozoites exhibited normal morphological features. Lower concentrations of 312.5 µg/mL and 625 µg/mL of the SE exhibited similar characteristics to the non-treated trophozoites. Regarding the brew, 25% showed similar characteristics to the non-treated trophozoites, while 50% showed trophozoites replication at 72 h but without significant difference to the 24 h mark. These results are consistent with an earlier study, which reported complete destruction of *A. castellanii* cysts and inhibition of encystation when exposed to *C. sinensis* SE, compared to the control. [12]

### Ultrastructural Changes in the Treated Encysting Trophozoites

**Fig. 3:**
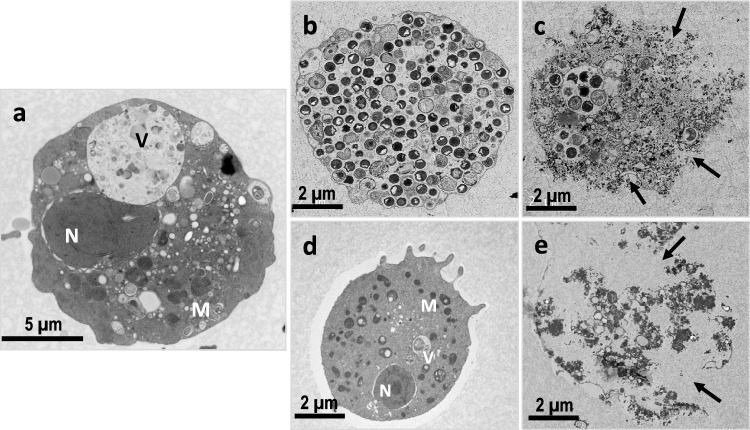
Representative TEM micrographs showing progressive destruction of *A. castellanii* cysts exposed to *C. sinensis* SE and brew. **a** Cyst in control encystation buffer; **b** Cyst exposed to 625 µg/mL of SE at 24 h shows increased condensation of the cellular contents; **c** Cyst exposed to 2500 µg/mL SE for 72 h showing loss of membrane integrity (arrow) with debris observed in the background. **d** Cysts exposed to brew at 24 h show loss of cellular membrane material. **e** At 72 h post exposure to brew, the cyst loses the cellular membrane integrity. *V* food vacuole, *M* mitochondria, *N* nucleus

TEM revealed extensive alterations of the internal structures of the treated cysts compared to untreated cysts (Fig. 3a). At 24 h post-exposure to *C. sinensis* SE, there was increased condensation of the cellular contents and an abnormal increase in the number of food vacuoles compared to the negative control (Fig. 3b). Cysts treated with *C. sinensis* brew showed a less intact membrane, fewer cytoplasmic vacuoles and condensation of the mitochondria, and abnormally condensed chromatin (Fig. 3d). At 72 h post-exposure to *C. sinensis* SE, the cysts lost their membrane integrity and ruptured, leading to an extensive expulsion of the cellular contents (Fig. 3c). Cysts treated with *C. sinensis* brew also showed marked loss of cellular membrane and depletion and expulsion of the cytoplasmic contents (Fig. 3e). These results are consistent with an earlier study where the extracellular morphological changes of complete destruction *A. castellanii* cysts and encystation inhibition when exposed to *C. sinensis* SE when compared to the control. [12]

### C. sinensis Extract Compromises the Nuclear Integrity of Trophozoites

**Fig. 4:**
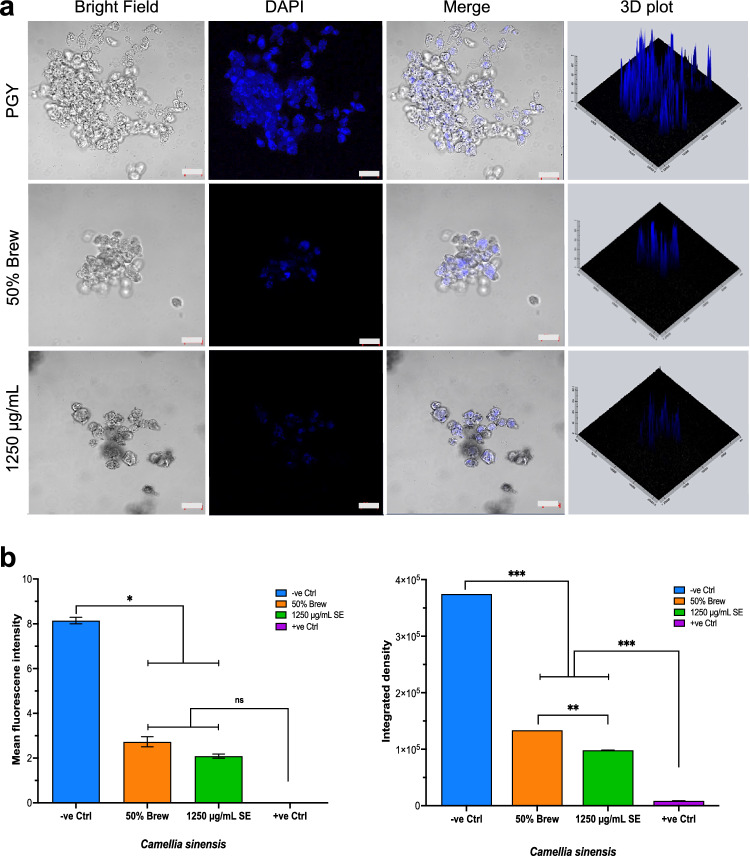
Examination of the nuclear integrity of trophozoites using DAPI staining. **a** Staining using DAPI shows a qualitative increase in the fluorescent intensity between the PGY-treated trophozoites and those treated with 50% *C. sinensis* brew and 1250 µg/mL *C. sinensis* SE. In the bright field and merged images, the treatment groups show less trophozoite clusters in comparison to the negative control (PGY). The 3D plots show clear qualitative differences between the fluorescence intensities. Scale bars, 20 µM. **b** Quantitative analysis of fluorescence expression after DAPI staining showing a significant increase in the mean fluorescent intensity between PGY-treated (-ve control) and *C. sinensi*s (brew and SE) treated trophozoites, and no significant difference between the positive (+ ve) control and *C. sinensis* forms (brew and SE). There were also significant increases and decreases in the integrated fluorescence density of the -ve and + ve controls, respectively, when compared to *C. sinensis* forms (brew and SE). The bar representing the + ve control is not shown as the trophozoites were all destroyed. (*p ≤ 0.05; ***p ≤ 0.001; *ns* non-significant)

The effect of *C. sinensis* SE and brew on the integrity of the trophozoites nucleus, using confocal imaging with 4′,6′-diamidino-2-phenylindole (DAPI) was marked, with significant increase in the fluorescence intensity of the control trophozoites when compared to trophozoites exposed to 1250 µg/mL SE and 50% *C. sinensis* brew for 24 h (Fig. 4a). The fluorescence micrographs showed qualitative disruption and loss of integrity of the nuclear envelope of trophozoites, which was associated with chromatin condensation (pyknosis) and DNA fragmentation (karyorrhexis). Volcano plots showed the quantitative difference between the fluorescence intensities. In the bright field and merged images, 1250 µg/mL SE showed less trophozoite clusters in comparison to 50% *C. sinensis* brew, and much fewer clusters when compared to the negative control. For DAPI, one-way ANOVA of the mean fluorescence intensity revealed a significant main effect of treatment (*p* = 0.0175). Post-hoc comparisons between the *C. sinensis* brew/SE forms and the negative control showed significant decrease in the mean fluorescence (*p* < 0.05) Further comparisons showed that there was no significant difference between the mean fluorescence intensity of the *C. sinensis* 1250 µg/mL SE and 50% brew and the positive control (*p* > 0.05). (Fig. 4b). For integrated fluorescence density analysis, a significant main effect of treatment was detected (*p* = 0.0005). Post-hoc comparison between the negative control and the *C. sinensis* forms (brew/SE) also showed significant increase of the integrated fluorescence density. Further comparison showed that there was a significant decrease in the integrated fluorescence density of the positive control when compared with the *C. sinensis* forms (*p* < 0.05).

**Fig. 5:**
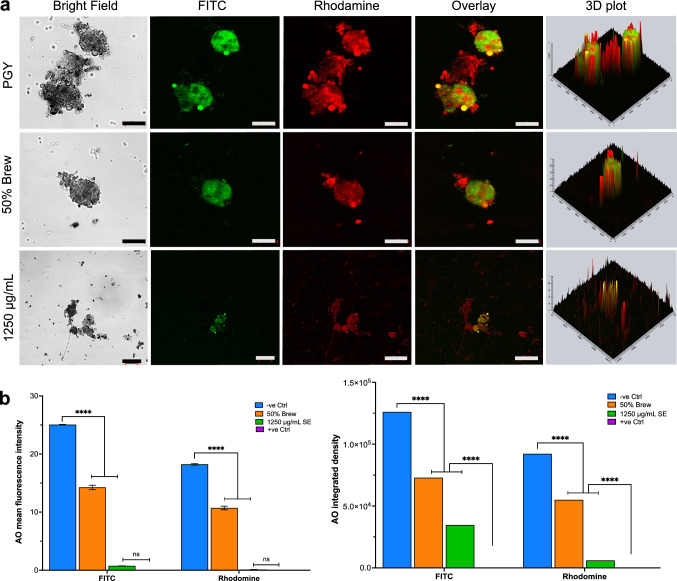
Examination of nuclear integrity of trophozoites using the Acridine orange (AO) staining. **a** Staining with AO shows a qualitative increase in the fluorescence intensity between trophozoites treated with PGY compared to *C. sinensis* 1250 µg/mL and 50% brew. The viability of trophozoites was greatly diminished as shown by the low fluorescence intensity measured after 24 h exposure. Scale bars, 10 µM. **b** Quantitative analysis of fluorescence expression after AO staining shows a significant increase in the mean fluorescent intensity between negative control (-ve control) and *C. sinensis* SE/brew, and no significant difference between the positive control (+ ve control) and SE. There were also significant increases and decreases in the integrated fluorescence density of the -ve and + ve controls, respectively, compared to *C. sinensis* SE. The bars representing the + ve control are not shown as the trophozoites were all destroyed. (****p ≤ 0.0001, *ns* non-significant)

Further analysis of the effect of *C. sinensis* SE and brew on the integrity of the trophozoite nucleus was performed using AO staining and confocal imaging. The negative control showed characteristic yellow-to-reddish orange spots overlaying larger greenish spherical structures with a polygonal arrangement of reddish spots and strands connecting the green spheres. Although similar structures were observed with the brew form, they appeared less and for SE, these structures appeared much shrunken. Treatment with acridine orange (AO) dye also showed a significant decrease of the fluorescence intensity with 1250 µg/mL of SE and 50% of brew within 24 h post-exposure compared to negative control. The sparse fluorescence detected for both filters and the overlay indicate that at 24 h post-treatment, 1250 µg/mL *C. sinensis* SE and 50% brew renders *A. castellanii* trophozoites less viable, leaving sparse nucleic acids (DNA/RNA) available for interaction with the dye (Fig. 5a) when compared to the negative control.

For AO dye, mean fluorescence analysis with two-way ANOVA revealed a significant main effect of treatment (*p* < 0.0001). Post-hoc comparisons showed that there was no significant difference between the mean fluorescence intensity of the positive control and the *C. sinensis* forms SE/brew (1250 µg/mL and 50%) (*p* > 0.05) for both FITC and rhodamine channels. For integrated fluorescence density analysis, two-way ANOVA revealed a significant main effect of AO dye (*p* < 0.0001), *C. sinensis* SE (*p* < 0.0001), and AO dye x *C. sinensis* SE inter-action (*p* < 0.0001). Post-hoc comparisons showed that there was a significant decrease in the integrated fluorescence density of the *C. sinensis* forms SE/brew when compared with the negative control (*p* < 0.05). The same results were observed when the positive control was also compared with the *C. sinensis* SE (Fig. 5b), there was significant increase of the integrated fluorescence density of *C. sinensis* forms SE/brews when compared to the negative control (*p* < 0.05). The decrease in integrated fluorescence density of *C. sinensis* SE and brew also corroborates the previous results that *C. sinensis* inhibits the growth and replication of *A. castellanii* trophozoites.

### FTIR Microspectroscopy Reveals Chemical Changes in the Treated Trophozoites

**Fig. 6:**
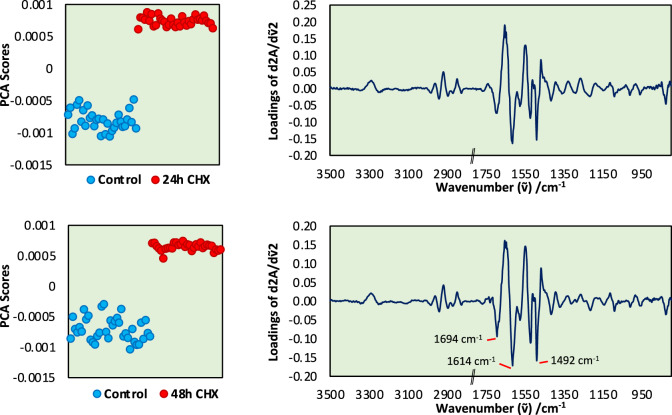
Principal component analysis (PCA) of trophozoites treated with Chx for 24 h and 48 h. PCA score plots showing the trophozoites’ spectra grouped together reflecting the distinct difference in the chemical composition of Chx-treated trophozoites and control trophozoites. The PC loadings describe what variants contribute to the grouping

The PCA results of the pair-wise comparison between the negative control and trophozoites treated with Chx at 24 h and 48 h are shown in Fig. 6. Near-identical PC1 (> 90% variance) scores and loading plots were observed for both time points with clear differences be-tween the Chx-treated and untreated (control) samples. We detected a characteristic peak at 1492 cm^−1^ for Chx, suggesting a high concentration of Chx inside trophozoites. Some of the peaks highlighted in the loading spectrum (1694 cm^−1^ and 1614 cm^−1^) can be attributed to alteration in protein secondary structure, with the treated trophozoites showing a higher content of beta-sheet protein.

**Fig. 7:**
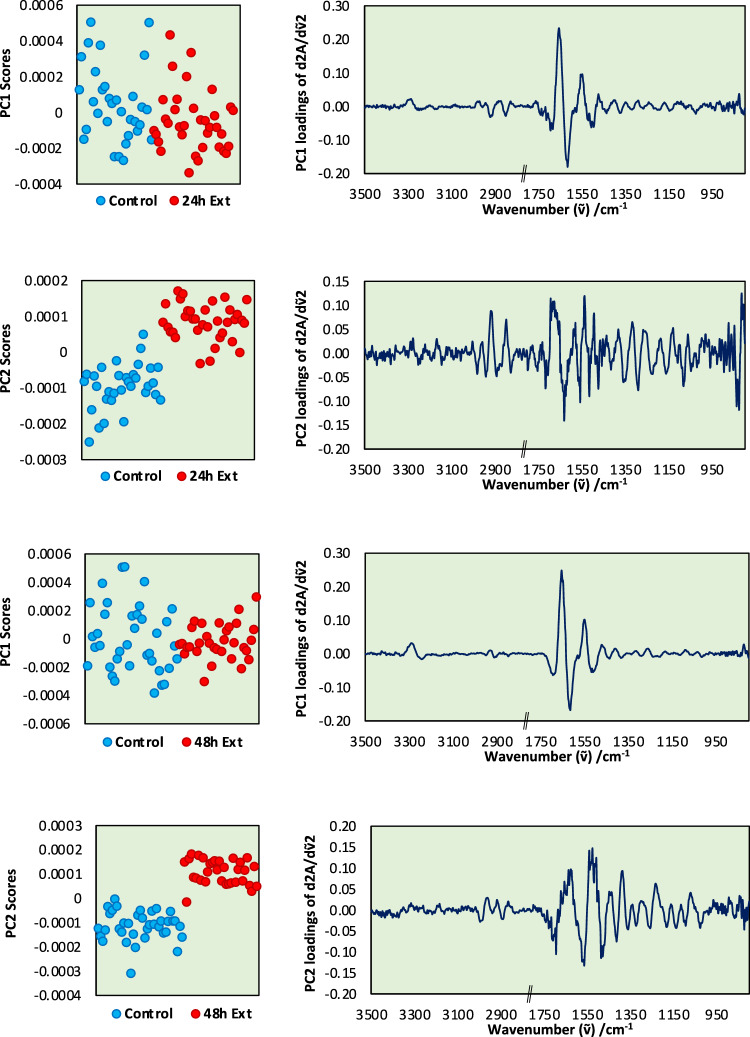
Principal component analysis (PCA) of trophozoites treated with *C. sinensis* SE. PC1 and PC2 plots of control trophozoites compared with trophozoites treated with *C. sinensis* SE for 24 h (**a**–**b**) and 48 h (**c**–**d**), respectively

Chx analysis has highlighted that FTIR microspectroscopy is sensitive and able to reveal the overall chemical changes in trophozoites when exposed to Chx. Next, the PCAs of FTIR data of single trophozoites (*n* = 35) comparing control and *C. sinensis* SE treatment are shown in Fig. 7. At 24 h after treatment, PC1 (48% variance) revealed some significant differences in protein amide I and II region (*p* < 0.05). PC2 (14% variance, which represents the 2nd most important variance in the data that is orthogonal to PC1), contrarily, clearly separated the control and treatment groups (*p* < 0.0001) and complex changes are indicated by the multiple peaks over the analyzed spectral regions. The PC1 (54% variance) of the 48-h treatment revealed variations in the protein amide I and II peaks in the trophozoites, which was not only attributed to the effect of treatment, but the duration of the treatment (*p* > 0.05), suggesting natural variations in the protein content between individual trophozoites. Importantly, PC2 (19% variance) was related to the treatment (*p* < 0.0001) and the spectral changes were focused in the 1800–900 cm^−1^ region where absorbances were associated with protein (amide I at 1690–1640, amide II at 1580–1500 cm^−1^ and amide III at ~ 1250–1220 cm^−1^), lipids (1745 and 1460 cm^−1^), nucleic acids (1714, 1235 and 1080 cm^−1^) carboxylate (~ 1600 and ~ 1400 cm^−1^), phosphate (~ 1240 and ~ 1080 cm^−1^) and carbohydrates (1300–1000 cm^−1^) metabolites as well as glycogen (1150, 1080 and 1030 cm^−1^).

**Fig. 8:**
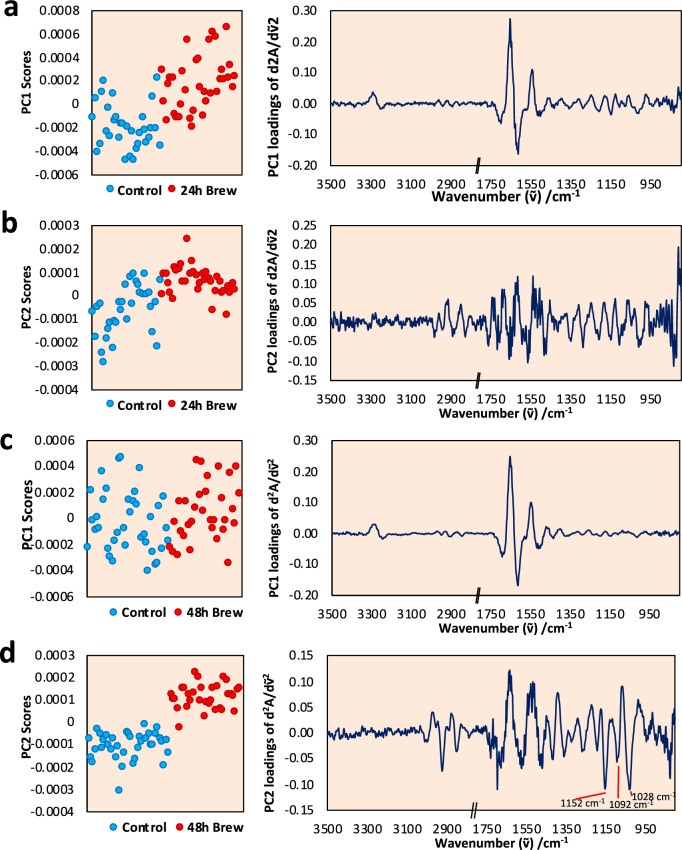
Principal component analysis (PCA) of trophozoites treated with *C. sinensis* brew. PC1 and PC2 plots of control trophozoites compared with trophozoites treated with *C. sinensis* brew for 24 h (**a**–**b**) and 48 h (**c**–**d**), respectively

The PCAs of FTIR data of single trophozoites (*n* = 35) comparing control and *C. sinensis* brew-treated trophozoites are shown in Fig. 8. Both PC1 (60.0% variance) and PC2 (8% variance) produce statistically significant differences between the control and the treated samples *(p* < 0.0001). For the 24 h treatment, PC1 revealed the difference in the amide I and II regions, suggesting that some of the treated trophozoites have changes in the protein composition when treated with *C. sinensis* brew. PC2 produced a loading plot that suggests some trophozoites have also produced biochemical changes. At 48 h after treatment, a similar PC1 (46.4% variance) loading plot was produced but the score plot did not associate this to the effect of the treatment (*p* > 0.05). The changes in the protein amide I and II peaks are possibly related to bio-logical variations between trophozoites. However, PC2 (13.8% variance) clearly differentiated between the control and treatment groups (*p* < 0.0001) with three characteristic peaks (1152 cm^−1^, 1092 cm^−1^ and 1028 cm^−1^) associated with glycogen absorbance bands which were elevated in the treated trophozoites.

## Discussion

Recent studies showed that brews and SE of *C. sinensis* possess anti-acanthamoebic activity [11, 12]. In this study, we examined and compared the effects of *C. sinensis* SE and brew on *A. castellanii*, and to also clarify its mechanism of action with regards to the interaction of their components to nucleic acids and proteins. We achieved this by examining the effects of exposure to *C. sinensis* on the structure, nuclear integrity, and chemical fingerprinting of *A. castellanii* using fluorescent dyes and FTIR microspectroscopy. The earlier studies used light microscopy, TEM, and colorimetric assays. We employed a range of methods to characterize the effects of *C. sinensis* SE and brew on the structure and function of *A. castellanii*. TEM showed that exposure of *A. castellanii* to *C. sinensis* SE resulted in considerable signs of cellular damage, including rupture of the nuclear and plasma membrane, nuclear fragmentation, and a shrunken cytosol. The significant loss of cell wall integrity observed in the studies, might be attributed to pore formation, membrane permeabilization, which leads to loss of ions and metabolites, interruption of vital functions, and ultimately leading to cell death. A close resemblance to *C. sinensis* SE effects were also seen in the effect of *C. sinensis* brew to *A. castellanii*.

Significant ultrastructural changes were observed in the trophozoites, and cysts exposed to both forms of *C. sinensis*. Disruption of the plasma membrane of trophozoites suggests cell damage brought about by some factors, especially the inhibition of cellulose, galactose and protein synthesis [12, 21]. *A. castellanii* possesses the ability to differentiate to cysts when exposed to chemotherapy and hyperosmotic conditions [22, 23], however their differentiation seemed to be inhibited by *C. sinensis* SE and brew.

The DAPI staining assay was used to evaluate the nuclear integrity based on the appearance of nuclear morphology (e.g., condensation and fragmentation). When compared to the negative control, and the brew, SE-treated trophozoites exhibited a much fainter DAPI fluorescence, indicating pyknosis, nuclear fragmentation, suggesting some forms of cellular damage of the treated trophozoites. The condensation or fragmentation of the nuclear chromatin suggests cell death of *A. castellanii* [24, 25]. The fluorescence intensity reduction, which indicates a reduction of *C. sinensis* treated trophozoites, is proportional to the smaller quantity of DNA and therefore the DNA synthesis, and in turn the replication of trophozoites. The volcano plots of fluorescence intensity confirmed a progressive nucleic acid reduction from the control compared to *C. sinensis* brew and SE, with no fluorescence detected with Chx treatment due to the complete destruction of trophozoites.

To further confirm that *C. sinensis* induced structural changes in the nucleus in trophozoites, AO-based fluorescence staining was used. The AO assay was used previously to analyze corneal scrapes from an AK patient and to detect *A. castellanii* trophozoites in axenic cultures [26–28]. Those studies revealed shrinking of treated trophozoites and cysts with a condensation of cytoplasm and chromatin, indicative of apoptotic trophozoites and/or cysts. In the present study, based on AO’s intercalation with DNA and electrostatic attractions with RNA, the results showed a reduction in DNA and RNA quantities, with fluorescence intensity of trophozoites treated with the SE being significantly low, compared with fluorescence intensity of trophozoites in the negative control and treated with brew The volcano plots representing AO fluorescence intensity con-firmed a nucleic acid reduction for the SE and brew. This result suggests significant nucleic acid damage caused by *C. sinensis* which was more pronounced both visually and statistically in the SE when compared to the brew.

FTIR data from treatment with *C. sinensis* brew showed differences in the amide I and II regions 24 h after treatment, suggesting that some of the treated trophozoites had changes in their protein structure and/or composition [29]. The alterations of proteins in the amide I and II regions might also suggest that the trophozoites underwent oxidative stress, which can lead to apoptosis [30]. There were differences between the control and brew spectra regarding the peaks that are negatively correlated with the amount of RNA [31] at 914, ~ 974 and 1118 cm^−1^ with only glycogen absorbance bands being elevated in trophozoites treated with brew with characteristic peaks of 1152 cm^−1^, 1092 cm^−1^ and 1028 cm^−1^. These bands were absence at 24 h treatment, but present in 48 h treatment. This indicates that the presence of *C. sinensis* brew inhibited the proliferation of the trophozoites confirmed by the reduction of RNA absorbance bands [32]. For *C. sinensis* SE-treated trophozoites, there was a close similarity to the changes observed in amide I and II regions, which extended to the complex changes in the phosphate/glycogen band region. These spectral changes are confirmed with the bioassays that are originated from the *C. sinensis* SE which inhibited *A. castellanii* trophozoites proliferation and promoted trophozoite death.

## Conclusions and Prospects

We characterized and compared the effects of *C. sinensis* SE and brew on the structure and function of *A. castellanii*. *C. sinensis* SE caused considerable damage and disruption of the nuclear integrity in the trophozoites. FTIR microspectroscopy revealed the lack of amide I and II bands. These results suggest that *C. sinensis* SE and brew can disrupt the structures and vital functions in *A. castellanii*, including DNA integrity and protein synthesis. Our previous study showed that epigallocatechin-3-gallate and caffeine had promising anti-acanthamoebic activity [12], suggesting that *C. sinensis* constituents are good candidates for further investigation. Future studies should therefore examine the effect of *C. sinensis* constituents on the structure and function of *A. castellanii* and ascertain their pertinence for clinical evacuation, which may ultimately result in clinical use.

## Supplementary Information

Below is the link to the electronic supplementary material.

Supplementary file1 Fig. S1. The effect of flat CaF2 substrate versus ZnS lenses on the FTIR baseline spectrum. Randomly selected FTIR spectra of control trophozoites measured (a) on a flat CaF2 substrate and (b) in between two ZnS lenses. Both measurements were made with the same spectral resolution of 4 cm-1 and 64 scans. (PDF 428 KB)

## Data Availability

The original contributions presented in the study are included in the article and supplementary material, further inquiries can be directed to the corresponding authors.
