# Supplementary figures and images for: Morphological and Chemical Changes in the Trophozoites and Cysts of Acanthamoeba Castellanii Induced by Camellia Sinensis Extracts
Source: Acta Parasitol. 2025 Mar 3;70(2):63. doi: 10.1007/s11686-024-00941-9 (PMC11876251; doi:10.1007/s11686-024-00941-9)

**a**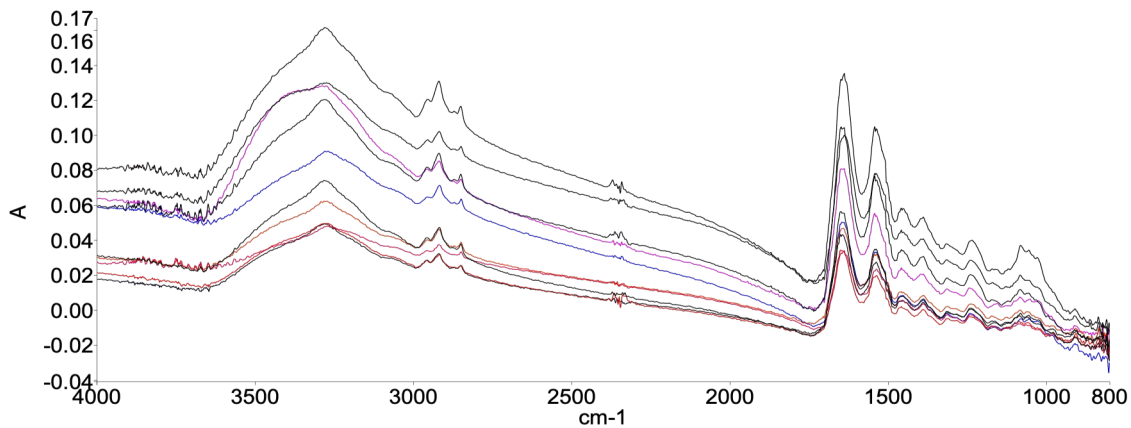**b**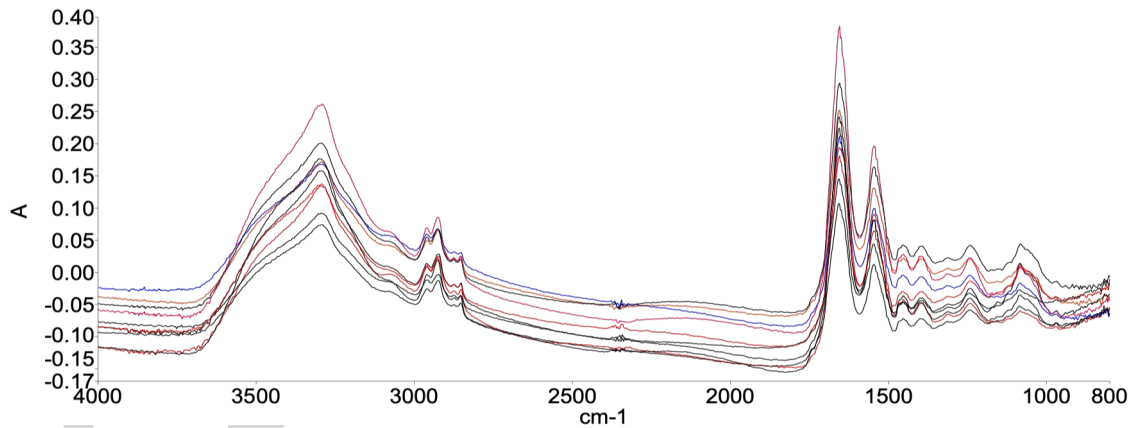

Supplement: Supplementary file 1 — Supplementary file1 Fig. S1. The effect of flat CaF2 substrate versus ZnS lenses on the FTIR baseline spectrum. Randomly selected FTIR spectra of control trophozoites measured (a) on a flat CaF2 substrate and (b) in between two ZnS lenses. Both measurements were made with the same spectral resolution of 4 cm-1 and 64 scans. (PDF 428 KB) [file 11686_2024_941_MOESM1_ESM.pdf]
